# Serum HDL-C level of Iranian adults: results from sixth national Surveillance of Risk Factors of Non-Communicable Disease

**DOI:** 10.1186/2251-6581-13-67

**Published:** 2014-06-16

**Authors:** Mostafa Hosseini, Iman Navidi, Mahmoud Yousefifard, Ramin Heshmat, Jalil Koohpayehzadeh, Fereshteh Asgari, Koorosh Etemad, Ali Rafei, Mohammad Mehdi Gouya

**Affiliations:** 1 Department of Epidemiology and Biostatistics, School of Public Health, Tehran University of Medical Sciences, Tehran, Iran; 2 Department of Physiology, School of Medicine, Tehran University of Medical Sciences, Tehran, Iran; 3 Chronic Disease Research Center, Endocrinology and Metabolism Population Sciences Institute, Tehran University of Medical Sciences, Tehran, Iran; 4 Endocrinology and Metabolism Research Center, Tehran University of Medical Sciences, Tehran, Iran; 5 Department of Community Medicine, Iran University of Medical Sciences, Tehran, Iran; 6 Saveh Medical University, Saveh, Iran; 7 Center for Disease Control, Ministry of Health and Medical Education, Tehran, Iran; 8 Department of Epidemiology, School of Public Health, Sahid Beheshti University of Medical Sciences, Tehran, Iran

**Keywords:** Serum HDL-C, Normal level, Iranian population

## Abstract

**Background:**

Reduced level of high-density lipoprotein-cholesterol (HDL-C) is shown to be in association with the risk of coronary artery disease (CAD), metabolic syndrome, and chronic renal disease. Lack of a national representative research for assessing the level of HDL-C among Iranian adults, which is essential for health policy makers, was the motivation for this study.

**Methods:**

HDL-C levels of 4,803 Iranian adults aged 25–64 years old were measured by sixth national Surveillance of Risk Factors of Non-Communicable Disease (SuRFNCD) in 2011. Data were entered into STATA 12 software and were analyzed using fractional polynomial model and other statistical methods.

**Results:**

In average, Iranian adult women had 5.8 ± 0.3 mg/dL higher HDL-C level than men. The analysis showed that the HDL-C levels will be changed at most 3 mg/dL from the age of 25 to 64 years. Furthermore, it was shown that approximately half of the men and one third of the women had HDL-C level less than 40 mg/DL. Also HDL-C level of more than 60% of the women was less than 50 mg/dL.

**Conclusions:**

High level of HDL-C among Iranian adults was shown in this study which can be a major reason of increasing incidence of heart diseases in Iran. Hence, formulating policy regulations and interventions in Iranian lifestyle to reduce HDL-C levels should be among top priorities for health politicians.

## Introduction

Epidemiological data have shown an inverse relationship between plasma levels of HDL-C and the risk of coronary artery disease (CAD), myocardial infarction and atherosclerotic vascular disease [1]. Besides, when plasma HDL level is reduced, an increased risk of future ischaemic heart disease (I.H.D) occurs [2]. The cardio-protective effects of HDL seem to be multiple. Removal of macrophage cholesterol from the plaques considered to be the major anti-atherosclerotic mechanism of HDL [3].

In addition, HDL-C is reported to be a main factor for metabolic syndrome by International Diabetes Federation (IDF) [4] and National Cholesterol Education Program (NCEP) [5] definitions. In both of them, reduced level of HDL-C (<40 mg/dL and <50 mg/dL in men and women, respectively) accompanied by other factors, is defined as having metabolic syndrome. Furthermore, a new study by Amy Rebecca Bentley *et al*. has demonstrated that reduced levels of HDL-C go along with chronic renal disease [6].

To date, just a few researches [7-9] have studied the HDL-C levels of adults Iranian population. Furthermore, all of these studies have reported the levels or prevalence of having low level among specific regions of the country. Adverse effects of HDL-C in coronary heart disease (CHD) and renal functions, suggests spotting the serum levels of HDL-C which can be beneficial in public health planning. Therefore, in this paper we intended to assess the HDL-C levels of Iranian adults and find the percentage of risk groups among them.

## Materials and methods

### Sampling procedure

Sixth National Surveillance of Risk Factors of Non-Communicable Disease (SuRFNCD) was conducted in 2011. Iranian dwellers aged between 6 to 70 years were participated in this study. A multistage sampling framework was used in which counties (or a combination of small adjacent counties) assumed as primary sampling units. Cities or villages were supposed as the secondary sampling units (SSU) and households were assumed as sampling listing unit. Finally, dwellers aged between 6 to 70 years were considered as the sampling elementary units. Consequently, one individual was selected using a KISH method from both age groups 6–54 and 55–70 years at each selected household.

The present survey was approved by the Board of Ethics Committee of the Iranian Center for Disease Control. A consent form was read by the interviewer and acceptance or refusal to participate was formally recorded. All procedures were conducted in accordance with the guideline of the Declaration of Helsinki.

From each adult individual (aged 25 years or older), 10 ml of venous blood were took in sitting position by trained laboratory technicians. The sampling was carried out after 10 to 12 hours of fasting. Cold boxes were used for transferring blood samples to referral laboratory centers. Enzymatic method (Parsazmun, Karaj, Iran) with the coefficient of variation of 5% was used to measure serum HDL-C. The results from double checking in a National Reference Laboratory which was a WHO collaborating center in Tehran on 10% of the blood samples from the laboratory sites showed the reliability of overall measurements.

### Statistical analysis

Data were entered into STATA 12 [10] statistical software. In this study, only data from adults aged between 25 to 64 years with recorded age, sex, and HDL-C level were used for further analyses. HDL-C levels were modeled for men and women using fractional polynomial method by age, separately. First, HDL-C measurements were transformed logarithmic in order to follow a normal distribution, and then the mean of the transformed values were modeled. Second, the standard deviation of the transformed values was also modeled by age. The desired centiles, then, calculated using back transformation of the mean, SD, and normal distribution values. Similarly, to obtain the centiles of the cut-off values, we calculated the z-scores based on mean and the SD of the values and computing the inverse of the z-scores. It is worth mentioning that as the sampling framework of the SuRFNCD 2011 was multistage, Complex Sampling Survey method was applied to all of the analyses by SVY command in Stata software.

## Results

In the year of 2011, 7,510 Iranian adults aged 25–64 were participated in the sixth national SuRFNCD. About 40.6% (3,048 persons) were male, 59.4% (4,461 persons) were female. Only the sex of one participant was not recorded. HDL-C level (mg/dL) of 4,803 adults (1,807 (37.6%) men, 2995 (62.4%) women, and one adult with unrecorded sex) was measured.

**Figure 1:**
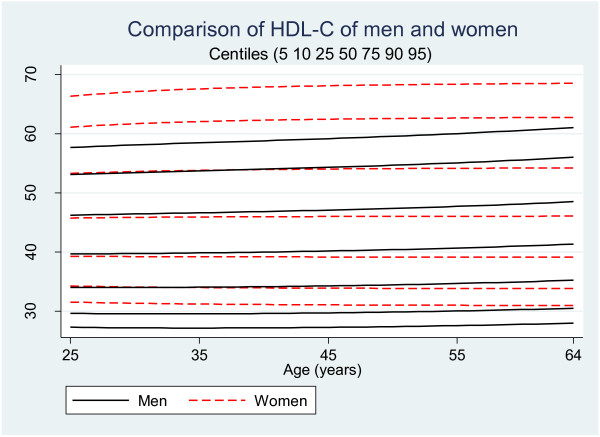
Comparison of HDL levels of Iranian men and women.

Figure 1 shows the modeled HDL-C levels for each sex by age. Average HDL-C level of women was significantly (5.8 ± 0.3 mg/dL) higher than men. This figure also shows that the level of HDL-C is not noticeably associated with the age and it hardly changes with the age of the subjects. Table 1 shows the average level for 50th, 75th, and 90th centiles by each age group and sex. This table suggests that HDL-C levels increase at most 3 mg/dL when the age increases from 25 to 64.

**Table 1:** **The average of 50**^th^, 75^th^ and 90^th^**percentiles of HDL-C of women and men according to age group**

**Age groups**	**Women**			**Men**		
	**50**^**th**^	**75**^**th**^	**90**^**th**^	**50**^**th**^	**75**^**th**^	**90**^**th**^
25-34	46	54	62	40	46	53
35-44	46	54	62	40	47	54
45-54	46	54	63	40	47	55
55-64	46	54	63	41	48	56

Since the low levels of HDL-C can be considered as a risk factor for both CHD and metabolic syndrome, Table 2 shows the percentage of Iranian population who are in risk based on cut-offs suggested by ATP III [11] and American Heart Association [12]. Having HDL-C <40 is classified as the risk for CHD in both women and men. Also, HDL-C <40 for men and HDL-C <50 for women is considered as a risk factor for metabolic syndrome. This table shows that a high percentage of Iranian population are categorized as individuals with low HDL-C that make them susceptible to hearth diseases.

**Table 2:** Percentage of low HDL risk factor for CHD and metabolic syndrome among Iranian population by age group

**Age group**	**Percentage of low HDL risk factor**			
	**CHD**		**Metabolic syndrome**	
	**Men (<40 mg/dL)**	**Women (<40 mg/dL)**	**Men (<40 mg/dL)**	**Women (<50 mg/dL)**
25-34	51%	28%	51%	65%
35-44	50%	28%	50%	64%
45-54	48%	28%	48%	63%
55-64	46%	28%	48%	63%

## Discussion

In this study, it has been shown that HDL-C level was considerably low for Iranian population. About half of the men and one third of the women had HDL-C less than 40 mg/dL. In addition, more than 60% of Iranian women had the level less than 50 mg/dL. This results show that Iranian population may be more prone to heart and metabolic diseases. Furthermore, it was shown that HDL-C levels do not change significantly by age and are higher in women than men of Iranian population.

To Date, some studies had researched the levels of HDL-C among Iranian population. All of these studies were carried out in specific regions, cities, and districts and there was no national representative study. The most well-known study has been performed by Azizi *et al*. [9] in Tehran lipid and glucose study. This study was carried out in district 13 of Tehran city between February 1999 and May 2000. The median of HDL-C level for most adult age groups was reported to be about 39 and 46 for men and women, respectively. Although their analysis was only executed in one district of Tehran city and they did not perform any statistical modeling, their reported levels were somewhat similar to our results. This similarity suggests that capital of the countries could also be a good representative for the HDL-C profile of a country, as the similar deduction has been shown by Hosseini *et al*. [13] when studying growth indexes of children.

Sharifi *et al*. [7] investigated the prevalence of low HDL-C concentrations among Iranian adults living in northwestern of Iran with age more than 20 years. Their findings showed that 63% of men and 93.3% of women had low HDL-C. In their analysis low HDL-C concentrations was defined as having <40 mg/dL and <50 mg/dL for men and women, respectively. These cut-off points are considered to be risk factors for metabolic syndrome [12]. In our study, we have found that about 50% of men and 64% of women were categorized as low HDL-C group which is noticeably far different than theirs that suggests existence different patterns of HDL-C in the country.

Another study carried out in Tehran by Hatami *et al*. [8] investigated the prevalence of low HDL-C which was defined as having HDL-C <35 mg/dL. It was shown that 5.4% of the participants had levels less than 35 mg/dL. Also, the mean ± SD of HDL-C level for the subjects was 41.7 ± 13.2 mg/dL. Combining the data from both males and females in our study showed that the mean ± SD of the subjects in our study was 45.3 ± 11.5 mg/dL. The rationale behind these differences could be the geo-location of the participants. Our analysis showed that although all of the provinces across the country had noticeably lower levels of HDL-C than other countries, the percentage of people who belong to risk groups varied from 25% to 75%, from one province to others (data not shown here).

Based on suggested cut-offs for HDL-C by ATP III and American Heart associations, our results showed that many adults are belong to the high risk groups for metabolic syndrome and CHD. These cut-offs have been achieved by studying on other populations (mainly people in United States), therefore, using such cut-offs might be misleading for other communities. However, a review study conducted by Ebrahimi *et al.*[14] showed that prevalence of metabolic syndrome and CAD are both reported as high in Iranian population by many studies. The least prevalence of metabolic syndrome among Iranian adults was observed by Sharifi *et al.*[15] which was 23.7%. In addition, a study performed by Heidari [16] suggested that HDL-C along with waist circumference is a good predicator for metabolic syndrome based on ATP III criteria. Therefore, it might be said that these cut-offs can be useful for predicting related coronary and metabolic diseases.

It was previously shown that transition from rural to urban lifestyle has negative effect on lipoprotein profiles of a community [17]. Our analysis, however, showed that difference between HDL-C levels of rural and urban people can be at most 3%.

Finding appropriate cut-offs needs long term cohort or longitudinal studies. However, this study was a cross-sectional study conducted in 2011. Therefore, to assess the suggested cut-offs by other nations, it is better to conduct a cohort study. Another limitation of the study was that the HDL-C level of Iranian population showed significant difference between provinces. There might be other important factors influencing this level; however, our data did not include such factors.

## Conclusion

Our study showed that HDL-C levels of Iranian population are considerably low. On the other hand, prevalence of heart diseases is increasing in Iran. Therefore, immediate policy regulations regarding controlling lipoprotein profiles and the consumptions of Iranian adults are needed.

## Competing interests

Authors declare that there is no conflict of interests.

## Authors’ contributions

MH, RH and MMG devised the study design. IN wrote the first draft. MH, IN and RH analyzed data. JK, FA, KE, AR, MY and MMG managed data collection. MH and RH were project coordinator and responsible for data management and analysis. All authors commented on the final manuscript and provided critical revisions. All authors read and approved the final manuscript.
